# The therapeutic potential of targeting tryptophan catabolism in cancer

**DOI:** 10.1038/s41416-019-0664-6

**Published:** 2019-12-10

**Authors:** Christiane A. Opitz, Luis F. Somarribas Patterson, Soumya R. Mohapatra, Dyah L. Dewi, Ahmed Sadik, Michael Platten, Saskia Trump

**Affiliations:** 1 DKTK Brain Cancer Metabolism Group, German Cancer Research Center (DKFZ), Heidelberg, Germany; 2 Neurology Clinic and National Center for Tumor Diseases, University Hospital of Heidelberg, Heidelberg, Germany; 3 Faculty of Biosciences, Heidelberg University, Heidelberg, Germany; 4 Division of Surgical Oncology, Department of Surgery - Faculty of Medicine, Public Health and Nursing, Universitas Gadjah Mada/Dr Sardjito Hospital, Yogyakarta, 55281 Indonesia; 5 DKTK Clinical Cooperation Unit Neuroimmunology and Brain Tumor Immunology, German Cancer Research Center (DKFZ), Heidelberg, Germany; 6 Department of Neurology, University of Heidelberg, Medical Faculty Mannheim, Mannheim, Germany; 7 Charité – Universitätsmedizin Berlin and Berlin Institute of Health, Unit for Molecular Epidemiology, Berlin, Germany

**Keywords:** Immunosurveillance, Cancer metabolism

## Abstract

Based on its effects on both tumour cell intrinsic malignant properties as well as anti-tumour immune responses, tryptophan catabolism has emerged as an important metabolic regulator of cancer progression. Three enzymes, indoleamine-2,3-dioxygenase 1 and 2 (IDO1/2) and tryptophan-2,3-dioxygenase (TDO2), catalyse the first step of the degradation of the essential amino acid tryptophan (Trp) to kynurenine (Kyn). The notion of inhibiting IDO1 using small-molecule inhibitors elicited high hopes of a positive impact in the field of immuno-oncology, by restoring anti-tumour immune responses and synergising with other immunotherapies such as immune checkpoint inhibition. However, clinical trials with IDO1 inhibitors have yielded disappointing results, hence raising many questions. This review will discuss strategies to target Trp-degrading enzymes and possible down-stream consequences of their inhibition. We aim to provide comprehensive background information on Trp catabolic enzymes as targets in immuno-oncology and their current state of development. Details of the clinical trials with IDO1 inhibitors, including patient stratification, possible effects of the inhibitors themselves, effects of pre-treatments and the therapies the inhibitors were combined with, are discussed and mechanisms proposed that might have compensated for IDO1 inhibition. Finally, alternative approaches are suggested to circumvent these problems.

## Background

Tryptophan (Trp) is the least abundant essential amino acid, and its supply in humans is exclusively covered by dietary intake. Trp is important not only for protein synthesis but also as a precursor for a variety of biologically active compounds. The majority of free Trp is metabolised via the kynurenine (Kyn) pathway, generating metabolites with crucial functions in neurotransmission and the regulation of immune responses.^1^ The first step of this Trp-degradation pathway can be catalysed by either indoleamine-2,3-dioxygenase 1 (IDO1), IDO2 or tryptophan-2,3-dioxygenase (TDO2). These enzymes catalyse the oxidative cleavage of the indole moiety of Trp that leads to the formation of *N*-formyl-l-kynurenine, which in a subsequent step is degraded to Kyn and further downstream metabolites.^1^

IDO1/2 and TDO2 modulate immune responses and promote cancer progression by mediating Trp deprivation and the production of metabolites along the Kyn pathway.^1–4^ The depletion of Trp has been reported to facilitate tumour immune escape through induction of regulatory T cells (Tregs),^5,6^ downregulation of the T-cell receptor ζ-chain (TCRζ) in CD8^+^ T cells,^6^ and the induction of the inhibitory receptors ILT3 and ILT4 on dendritic cells (DCs).^5^ Moreover, Trp catabolic products downstream of IDO1/2 and TDO2 regulate immune cell function and promote cancer progression through activation of the aryl hydrocarbon receptor (AHR).^1,7–10^

The importance of Trp catabolism in the induction of immune tolerance was initially established in the placenta, where IDO1 was shown to prevent rejection of the foetus.^11^ Such mechanisms responsible for self-tolerance are exploited by tumours to escape immunosurveillance. IDO1 mediates its immunosuppressive function by promoting the formation of Tregs and myeloid-derived suppressor cells (MDSCs), while suppressing the proliferation and function of effector T cells and natural killer (NK) cells.^12–14^ TDO2 has also been shown to decrease anti-tumour immune responses in a similar manner.^7,15^

Due to the role of Trp catabolism in promoting immune suppression, several small-molecule inhibitors targeting Trp catabolism have been developed and are currently tested in clinical trials.^1,16,17^ Moreover, clinical trials combining IDO1 inhibitors with other immunotherapies, such as anti-programmed cell death 1 (PD1) and anti-programmed cell death ligand 1 (PD-L1) immune checkpoint inhibitors, have elicited high hopes of a positive impact in the field of immuno-oncology by synergising to restore anti-tumour immune responses.^18–22^ The failure of the IDO1 inhibitor epacadostat in combination with pembrolizumab in a Phase 3 clinical trial in advanced melanoma, and other negative clinical results with IDO inhibitors,^23,24^ raises many questions.

This review will give a brief overview of Trp catabolism and the mechanisms involved in its immunosuppressive and tumour-promoting properties. In addition, it will detail specifics of inhibitors targeting Trp-degrading enzymes and will discuss downstream mechanisms that might have contributed to the failure of the IDO1 inhibitor epacadostat in clinical trials. Furthermore, we will suggest strategies that might circumvent mechanisms compensating for IDO1 inhibition.

## Trp-catabolising enzymes (TCE)

### IDO1 expression in normal tissues and in cancer

In line with studies implicating IDO1 in preventing the rejection of allogeneic foetuses,^11^ high IDO1 protein expression is found at the foeto–maternal junction during human pregnancy;^25,26^ it is also expressed in the epithelium of the cervical gland,^26^ in lung endothelial cells^27^ and in numerous lymphoid organs,^28^ particularly in lymph nodes, where it is enriched in activated DCs.^28^ In the majority of cases, IDO1 expression is increased in cancerous tissue compared with the untransformed tissue of origin;^29,30^ IDO1 protein has been detected in endothelial cells,^31^ immune cells,^32^ and in tumour cells themselves.^28,31,33–35^

High expression of IDO1 has been shown to correlate with poor patient prognosis in numerous cancer types.^1,22^ Cancer cells can either constitutively express IDO1^27,28^ or can be induced to do so by tumour-infiltrating immune cells that secrete inflammatory cytokines, such as interferon γ (IFN-γ).^36,37^ In some tumour types, such as breast cancer or renal cell carcinoma (RCC), however, IDO1 expression has been linked to improved survival,^31,38–40^ most probably due to IDO1 acting as a surrogate marker for immune infiltration in these cancers,^41^ which often translates to improved survival rates.^42^ Notwithstanding the improved survival associated with IDO1 expression in these cancers, inhibition of IDO1 would be expected to further increase survival rates by enhancing the immune response against the tumour.

### IDO1-mediated Trp catabolism and immunosuppression

Trp catabolism through increased IDO1 activity enables cancer progression by suppressing anti-tumour immune responses. High IDO1 expression has been shown to correlate with reduced levels of infiltrating CD3^+^ T cells, CD8^+^ T cells, CD57^+^ NK cells, B cells^43–47^ and increased levels of forkhead box P3 (Foxp3)^+^ Tregs^48–52^ in different cancer types. Accordingly, in breast cancer patients, the number of IDO1-expressing MDSCs is increased and their IDO1 expression is positively associated with Foxp3^+^ Treg cell infiltration in tumours and lymph node metastases.^53^ Moreover, IDO1 expression in melanoma cells was shown to promote immunosuppression by expanding, recruiting and activating MDSCs in a Treg-dependent manner.^54^

The concentration of Kyn in tumour tissue and in the plasma of cancer patients is also associated with immunosuppressive phenotypes. In patients with breast or colon cancer, the expression of the immune checkpoint component PD-1 on CD8^+^ T cells showed a strong positive correlation with the concentrations of Kyn in blood and tumour tissue.^55^ Collectively, these studies provide evidence of the role of Trp catabolism in promoting an immunosuppressive tumour microenvironment, thus confirming the importance of IDO1 as an immunotherapeutic target.

### IDO2 and its role in cancer

IDO2 was discovered relatively recently,^56,57^ and was described to be present in human liver, small intestine, spleen, placenta, thymus, lung, brain, kidney and colon. Similar to IDO1, IDO2 is also expressed in antigen-presenting DCs,^57^ although IDO1 and IDO2 expression and their regulation differ among different populations of DCs.^58^ Under physiological conditions, IDO2 does not appear to contribute significantly to systemic Trp catabolism,^59^ and Kyn levels in the serum of *Ido2*^–/–^ mice do not differ from those of wild-type mice.^60^ *Ido2*^–/–^ mice showed no overt phenotypes in embryonic development, haematopoietic cell differentiation or their immune cell profile, but, upon immune stimulation, these mice displayed defective IDO1-dependent Treg cell formation.^60^ In support of this observation, human DCs promote the generation of Treg cells through a mechanism dependent on IDO2.^58^ Similar to IDO1, IDO2 has also been reported to inhibit the proliferation of CD4^+^ and CD8^+^ T cells.^61^

In addition to its functions in immune cells (reviewed by Prendergast and colleagues^21,62^), IDO2 has been implicated in promoting proliferation, survival and migration of cancer cells through a mechanism involving NAD^+^ production in a murine model.^63^ Although analysis of gene expression data from the cancer genome atlas (TCGA) reveals that *IDO2* expression is not frequently upregulated in human cancer,^64^ *IDO2* has been shown to be expressed in primary gastric, colon and RCCs,^65^ as well as in pancreatic ductal adenocarcinoma (PDAC).^66–68^ In mouse models, IDO2 expression promoted the development of PDAC.^66,67^ Moreover, the presence of biallelic IDO2-inactivating single-nucleotide polymorphisms (SNPs)^57,68^ was significantly associated with an improved disease-free survival in response to adjuvant radiotherapy in human patients with PDAC,^66,67^ suggesting that stratifying PDAC patients based on *IDO2* genotype status could be beneficial for treatment decision-making.

### TDO2 and its role in cancer

Under physiological conditions, TDO2 is expressed primarily in the liver, where its activity regulates systemic Trp levels.^15,69,70^ Furthermore, TDO2 expression has been detected in neurons and has been implicated in the regulation of neurogenesis and anxiety-related behaviour.^71,72^ TDO2 resembles IDO1 in that it is often expressed at higher levels in cancer tissue than in untransformed tissue.^64,73^ Specifically, TDO2 has been reported to be expressed in malignant glioma, hepatic carcinoma, non-small cell lung carcinoma (NSCLC), RCC, ovarian carcinoma, breast cancer, bladder cancer, basal cell carcinoma and melanoma.^7,15,74–76^ In co-cultures of glioblastoma cells with mixed leucocyte reactions (as an assay of T-cell competence), tumour cell TDO2 expression suppressed T-cell proliferation via the production of Kyn.^7^ This immunosuppressive effect was also observed in vivo, as TDO2 expression inhibited immune infiltration into gliomas^7^ and prevented tumour rejection in immunised mice.^15^ In addition, triple-negative breast cancer cells were reported to induce death of CD8^+^ T cells through TDO2-derived Kyn.^77^ Given the pro-tumour effects of TDO2 expression, recent research focus has been directed towards understanding factors regulating TDO2 expression in tumour cells. Tumour necrosis factor receptor-associated factor 7 (TRAF7) and AKT1 mutations in skull base meningiomas increase TDO2 expression in these tumours enabling suppression of immune responses.^78^ Apart from mutations, oncoviruses also appear to modulate TDO2 expression. Merkel cell polyomavirus (MCPyV)-positive Merkel cell carcinoma cells for instance show lower expression of TDO2 than MCPyV-negative cells.^79^ In malignant glioma, TDO2 expression is regulated via a steroid-responsive FK506-binding protein 4 (FKBP52)-dependent pathway,^80^ by prostaglandin E2 (PGE_2_) through activation of prostaglandin E receptor-4 (EP4)^81^ as well as by the homeobox transcription factor HOXC10, which directly binds to TDO2 promoter regions.^82^ In turn, expression of TDO2 has also been reported to regulate signalling in oesophageal squamous cell carcinoma cells, where knockdown of TDO2 expression inactivates the epidermal growth factor receptor (EGFR) signalling pathway.^83^

### Mechanisms underlying the actions of Trp catabolism

#### Effects of Trp shortage

**Fig. 1:**
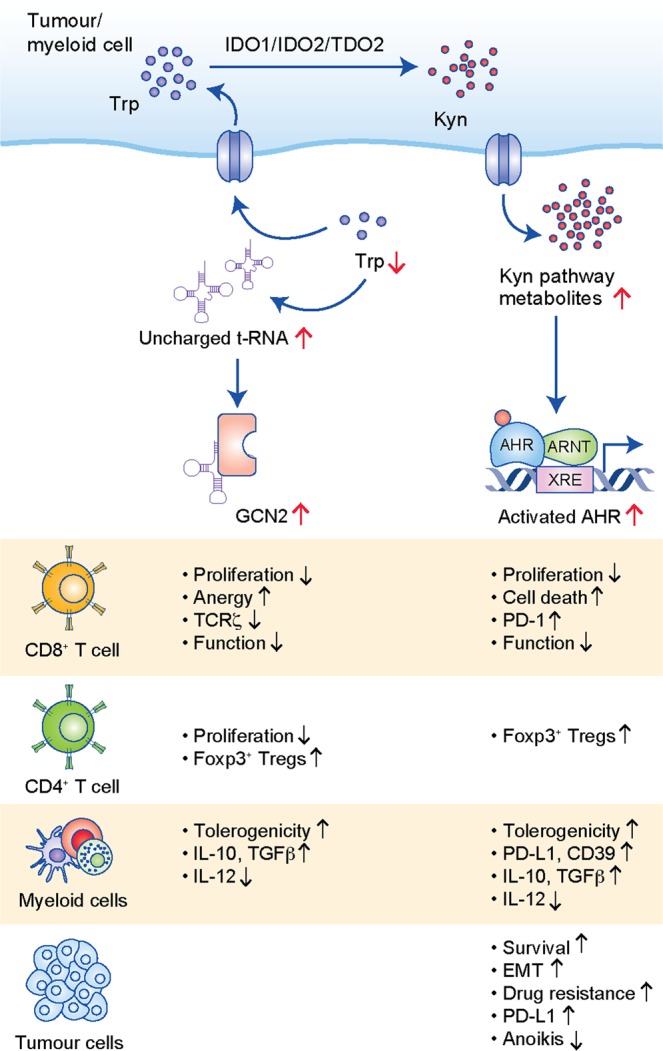
Mode of action and effects of Trp catabolism on cells in the tumour microenvironment. Effects of Trp depletion and Trp metabolites on CD8^+^ T cells, CD4^+^ T cells, myeloid cells and tumour cells are shown.

Trp depletion leads to the activation of the  general control non-derepressible-2 (GCN2) kinase and consequent phosphorylation of the eukaryotic initiation factor-2 α (eIF2α), thus blocking protein synthesis (Fig. 1). In CD8^+^ T cells, activation of the GCN2 pathway has been reported to lead to an inhibition of proliferation and induction of anergy,^84^ as well as downregulation of the TCRζ chain, which impairs the cytotoxic effector function of these T cells.^6^ Decreased levels of Trp also modulate CD4^+^ T-cell proliferation by downregulating the expression of enzymes involved in fatty acid synthesis,^85^ as well as CD4^+^ T-cell function by promoting the conversion of CD4^+^CD25^–^ cells into CD4^+^CD25^+^Foxp3^+^ Treg cells through a GCN2-pathway-dependent mechanism.^6^ Myeloid cells are also regulated by GCN2 kinase in response to low Trp concentrations. Under these circumstances, DCs become tolerogenic by increasing the expression of the inhibitory receptors immunoglobulin-like transcripts (ILTs) ILT3 and ILT4—hampering their ability to stimulate CD4^+^ T cells and promotes the induction of CD4^+^CD25^+^Foxp3^+^ Treg cells.^5^ Furthermore, induction of the IDO1–GCN2 pathway in macrophages can lead to an immunosuppressive phenotype, with increased production of the regulatory cytokines interleukin (IL)-10 and transforming growth factor-β (TGFβ), as well as suppression of the inflammatory cytokine, IL-12.^86^ In cancer cells, however, activation of GCN2 by Trp depletion induces the expression of tryptophanyl-tRNA-synthetase, which protects the cells against Trp shortage.^87^

Despite this well-documented association between Trp catabolism and GCN2-pathway activation, little evidence from experimental models has supported this hypothesis. While GCN2 in T cells and DCs plays an important role in restricting autoimmunity in mouse models,^88,89^ GCN2 in T cells has recently been demonstrated to be irrelevant in suppressing anti-tumour immunity in B16 melanomas.^90^ Moreover, evidence also points to the existence of a GCN2-independent pathway in T cells that senses low amino acid concentration and promotes cell-cycle arrest.^91^ The mammalian target of rapamycin (mTOR) pathway appears to be a possible candidate as it has been described as an alternative pathway that senses Trp-deprived conditions.^92,93^ Insufficient Trp concentrations in the microenvironment have been reported to inhibit the mTOR complex 1 (mTORC1) pathway^92^ and to promote the induction of Foxp3^+^ Treg cells.^93^ Further investigation is required to understand the relevance of low Trp concentrations in vivo and the specific conditions under which reduced Trp levels promote immunoregulation through the GCN2 and/or the mTOR pathway.

#### Activation of the AHR by Trp catabolites

Trp catabolic products, such as Kyn^7,94^ and cinnabarinic acid (CA),^95^ regulate immune cells through activation of the AHR^1,9,10,96,97^ (Fig. 1). Additional Trp catabolites, such as kynurenic acid (KynA)^98^ and xanthurenic acid (XA),^75^ have also been shown to activate the AHR in cancer cells, implying a likely immunoregulatory role of these metabolites. Kyn-mediated AHR activation was shown to induce the death of CD8^+^ T cells^77^ and upregulate the expression of PD-1 in CD8^+^ T cells,^55^ as well as to stimulate the differentiation of CD4^+^CD25^+^Foxp3^+^ Treg cells.^94,99^ Differentiation of Foxp3^+^ Treg cells is also promoted indirectly following AHR activation through the generation of DCs with an immunosuppressive phenotype.^99–101^ These DCs increase the production of the anti-inflammatory cytokines TGF-β1 and IL-10, and lower the production of pro-inflammatory cytokines, such as IL-1β and IL-12.^99^ In macrophages, Kyn-mediated activation of AHR induces a tolerogenic phenotype by regulating the expression of immunosuppressive molecules such as PD-L1.^97^ Moreover, the Kyn–AHR pathway has been implicated in promoting the recruitment of tumour-associated macrophages (TAMs) into tumours. Mechanistically, AHR induces the expression of C–C motif chemokine receptor 2 (CCR2) in TAMs, which are then recruited in response to C–C motif chemokine ligand 2 (CCL2) generated by tumours.^97^ Moreover, in TAMs, AHR activation triggers an immunosuppressive phenotype by inducing the expression of the ectonucleoside CD39, which promotes dysfunctional tumour-infiltrating CD8^+^ T cells due to the production of adenosine in association with CD73.^97^ Interestingly, cancer cells exposed to carcinogens contained in tobacco smoke also evade immune surveillance by inducing the expression of the immune checkpoint molecule, PD-L1.^102^ Notably, knockdown of AHR in cancer cells decreased the percentage of immunosuppressive CD11b^+^PD-L1^+^ tumour-infiltrating cells, increased tumour-infiltrating CD4^+^ and CD8^+^ T cells and inhibited tumour growth in an immunocompetent orthotopic model of oral cancer.^103^ This suggests that AHR expression in cancer cells alone can promote immunosuppression.

Apart from modulating the functions of tumour-infiltrating immune cells, AHR activation in cancer cells also promotes the loss of cell–cell contact and adhesion, which enables epithelial–mesenchymal transition (EMT), by downregulating the expression of E-cadherin.^104^ In line, AHR activation in cancer cells also induces cell motility,^7,74,75,105^ promotes cells survival^7^ and resistance to anoikis.^74^ Furthermore, AHR activation was shown to promote resistance against several cancer drugs, including BRAF inhibitors (BRAFi),^66^ tyrosine kinase inhibitors targeting the EGFR^106^ and chemotherapeutic agents.^107–109^ Recently, Kyn-mediated AHR activation was reported to promote proliferation of MYC-dependent cancer cells.^110^ The AHR pathway therefore constitutes a key immunoregulatory and cancer-promoting mechanism downstream of Trp catabolism.

### Other cancer-promoting effects of Trp degradation

Several lines of evidence point to a role for IDO1 in blood vessel formation. Compared with wild-type mice, mice with a genetic ablation of *Ido1* had a significantly reduced density of pulmonary blood vessels.^111^ Furthermore, neovascularisation in mouse models of oxygen-induced retinopathy and lung metastasis was significantly reduced in mice lacking *Ido1*.^112^ In line with the cell motility-promoting effects of Trp catabolites mediated via AHR activation (see above), the expression of Trp-catabolic enzymes has been shown to affect metastasis formation. Downregulation of IDO1 decreased lung cancer cell motility, while overexpression of IDO1 enhanced it.^113^ Consistent with this result, IDO1 promoted metastasis formation in preclinical models of breast and lung cancer.^111–114^ IDO1 overexpression in human lung cancer cells increased metastasis formation in immune-deficient mice,^113^ while IDO1 knockdown or genetic ablation decreased metastasis formation in mouse models of breast carcinoma.^111,114^ Of clinical relevance, the degree of IDO1 expression in cancer tissue has been reported to be positively associated with the occurrence of distant metastases in hepatocellular cancer,^115^ liver metastases in colorectal cancer^43^ and nodal metastases in endometrial carcinoma.^45^ Recently, IDO1 has been reported to be involved in the maintenance of pluripotency of human embryonic stem cells by promoting glycolysis through increase of the NAD^+^/NADH ratio,^116^ and it remains to be investigated if this phenomenon also has implications for cancer stem cells.

### Trp catabolism in preclinical models

Constitutive IDO1 expression is commonly observed in human tumour cells but is hardly present in mouse cancer cells^27^ (and own unpublished observations), which complicates preclinical studies investigating the effects of IDO1 in cancer, especially as syngeneic models are essential to investigate the immune effects of IDO1. *Ido1* has therefore been overexpressed in murine cancer cells to investigate its in vivo functions, or ablated from mice to investigate the effect of host *Ido1* on tumour development and progression. In mouse models of B16 melanoma^54^ and mastocytoma,^117^ *Ido1* overexpression promoted tumour growth through mechanisms involving inhibition of tumour-specific T cells^54,117^ and expansion of MDSCs within the tumour microenvironment.^54^ Although *Ido1* deficiency did not alter the size and number of colon tumours in Apc(Min/+) mice compared with wild-type mice, it increased the expression of pro-inflammatory cytokines in the tumour microenvironment while the number of Foxp3^+^ Treg cells was decreased.^118^ In a model of liver cancer, *Ido1* deficiency decreased the incidence and multiplicity of hepatocellular carcinoma and reduced infiltration of Foxp3^+^ Treg cells into the livers.^119^ *Ido1* ablation reduced tumour burden and infiltration of the tumours by MDSCs and PD-1-expressing CD8^+^ T cells in a mouse model of lung cancer.^120^ Furthermore, in a melanoma mouse model, knockout of *Ido1* slightly enhanced the therapeutic efficacy of anti-PD-1 /PD-L1 and anti-CTLA-4.^121^

The regulation of tumoural *Ido1* expression in mouse models can only be studied if endogenous *Ido1* levels are present and regulated in a similar manner as they are in the corresponding human tissues and cells. As *Ido1* is scarcely expressed in murine tumour cells, analysis of the effects of tumoural IDO1 expression is often only possible in mice implanted with human tumour cells, and reconstitution with human allogeneic lymphocytes has been employed for the analysis of the resulting immune effects.^122^ However, even in mouse cells expressing *Ido1*, its regulation can differ substantially from that of human IDO1 in the same cell types. Significant differences between IDO1 expression and regulation have, for example, been observed in human and murine mesenchymal stromal cells (MSCs).^123–125^ Similar to the case for IDO1, preclinical murine tumour models usually also lack TDO2 expression, distinguishing them from human cancers. Accordingly, strategies similar to the ones described above for Ido1 have also been employed to circumvent this issue. Injection of mice, immunised against a major antigen of tumour P815 cells, with murine TDO2-overexpressing P815 cells led to the development of tumours. In this model, TDO2 suppressed anti-tumour immune responses while the control P815 tumour cells were rejected.^15^ In summary, these issues render the preclinical evaluation of the function and regulation of Trp-degrading enzymes in murine in vivo models very challenging; hence, the analysis of human tissues and cells appears to be pivotal.

### IDO1 inhibitors in clinical trials

Numerous studies have supported the notion that IDO1-mediated Trp catabolism promotes tumour progression,^41,126–128^ thus making IDO1 inhibitors attractive targets for clinical development. As IDO1 is expressed at low levels in normal tissues but is upregulated in tumours,^64,73^ inhibition of IDO1 would not be expected to cause major adverse effects. This theory is supported by the fact that *Ido1*-knockout mice do not show an overt phenotype across different mouse strains, unless challenged by the Toll-like receptor ligands.^129^ Moreover, preclinical data indicated that IDO1 inhibition could synergise with immune checkpoint blockade (ICB).^19,121,130–132^ In line, clinical data suggest that IDO1 might be induced by immune checkpoint inhibition, as the plasma Kyn:Trp ratio was enhanced upon therapy with an anti-PD-1 antibody in sarcoma patients.^133^ IDO1 inhibitors have thus sparked great interest and various compounds are currently being tested in clinical trials, mainly as an adjunct to other treatment modalities such as chemotherapy and immunotherapy.^16,22,134^ Patents on IDO1 inhibitors have been comprehensively reviewed by Cheong et al.^16^

#### Epacadostat

Epacadostat (INCB024360), developed by Incyte Corporation, is an IDO1 inhibitor that competes with Trp for IDO1 binding by forming a direct bond with the haem iron atom of IDO1.^21,22^ It was the most advanced IDO1 inhibitor in clinical studies, both as a single agent and in combination with anti-PD-1, anti-PD-L1 or anti-CTLA-4.^18,24,135–137^ Epacadostat boosts anti-tumour effects by enhancing T-cell and NK-cell proliferation and function.^138^ In a preclinical study, epacadostat synergistically enhanced the inhibitory action of anti-PD-L1, anti-PD-1 and anti-CTLA-4 on melanoma growth.^139^ Multiple clinical trials with epacadostat, ranging from Phase 1 to 3, have been performed in solid tumours and haematological malignancies.^140^ A Phase 1 clinical trial of epacadostat confirmed its safety and 80–90% IDO1 inhibitory activity measured in plasma at doses ≥100 mg twice daily as a single agent. In seven out of 52 patients, stable disease lasting ≥16 weeks was observed.^141^ The majority of clinical trials of epacadostat have investigated its effect as an adjunct to the anti-PD-1 antibody pembrolizumab, with or without additional treatment modalities.^1,134^ For the treatment of advanced melanoma, metastatic NSCLC, RCC, urothelial carcinoma and head and neck squamous cell carcinoma several of these trials reached Phase 3.^140^ However, in the Phase 3 ECHO-301 trial, the combination of epacadostat with pembrolizumab failed to meet the primary endpoint of progression-free survival in advanced melanoma.^24^ Consequently, the Phase 3 clinical trials of epacadostat combined with immune checkpoint inhibition were either suspended or turned into Phase 2 trials with randomisation.

#### BMS-986205

BMS-986205 is an orally administered, irreversible IDO1 inhibitor, which binds to the apo-IDO1 protein lacking haem.^1,21,22^ Bristol-Myers Squib evaluated the therapeutic efficacy of BMS-986205 in combination with nivolumab, another anti-PD-1 antibody, in several advanced cancers (NCT02658890), metastatic or non-resectable melanoma patients (NCT03329846), NSCLC (NCT03417037) and head and neck cancer (NCT03386838). Preliminary results from 42 patients had confirmed the potency of BMS-986205 in reducing plasma Kyn levels by more than 60% and tumoural Kyn by up to 90% at 100 or 200 mg orally once daily,^142^ but Bristol-Myers Squib halted its three Phase 3 trials of BMS-986205 in combination with nivolumab in response to the failure of epacadostat in combination with pembrolizumab. However, patients continued to be recruited into Phase 1/2 clinical trials with BMS-986205, including those in combination with immune checkpoint inhibitors. At this point no further results of these trials are available.

#### Indoximod

Also known as 1-methyl-d-tryptophan (D-1-MT), indoximod, in contrast to direct IDO1 enzymatic inhibitors, acts as an IDO1-pathway modulator.^143^ Similar to Bristol-Myers Squib’s BMS-986205, NewLink Genetics did not commence its Phase 3 clinical trial of indoximod combined with pembrolizumab or nivolumab for patients with advanced melanoma. Indoximod showed favourable bioavailability and tolerability in cancer patients at doses up to 2000 mg orally twice/day in combination with docetaxel for advanced solid tumours (NCT01191216)^144^ in a Phase 1 clinical trial and achieved a best response of stable disease for more than 6 months in five out of 48 patients.^145^ However, the combination of indoximod and taxane (NCT01792050), studied in a Phase 2 trial in patients with metastatic breast cancer, failed to meet its endpoints regarding progression-free survival, overall survival or objective response rate.

#### Navoximod

Another IDO1 inhibitor developed by NewLink Genetics, navoximod/NLG919/formerly GDC-0919—which also shows inhibitory activity against TDO2—showed favourable pharmacokinetic activity and a reduction of plasma and tissue Kyn concentrations of up to 50% in mice.^146^ Navoximod binds directly to the haem iron atom of the IDO1 enzyme.^21,22^ When tested as a single agent in multiple solid tumours in humans, navoximod was well tolerated at doses of up to 800 mg twice daily. Navoximod only transiently decreased plasma Kyn levels by 25–30% 2–4 h after drug intake, there were no objective responses, and the rate of stable disease was 12/22 (68%).^147^ Despite showing IDO1 inhibitory activity, the combination of navoximod with the anti-PD-L1 antibody atezolizumab showed no clinical benefit in a Phase 1 study of 157 patients with advanced solid tumours.^23^ In line, a Phase 2 trial of 61 patients with advanced solid tumours only yielded a 10% response rate, most of which were only partial responses (NCT02471846).

#### EOS-200271

EOS-200271, formerly also known as PF-06840003, is an IDO1 inhibitor developed by iTeos Therapeutics, which displays non-competitive kinetics with respect to Trp and does not bind to the haem iron atom.^148^ Preclinical studies of EOS-200271 showed an 80% reduction in intra-tumoural Kyn levels and inhibition of tumour growth in multiple syngeneic mouse models, both as a monotherapy and, with an enhanced efficacy, in combination with PD-L1 blockade (Avelumab).^19,149^ In GL261 murine glioma, EOS-200271 demonstrated excellent central nervous system (CNS) penetration and synergistic effects in combination with PD-1/PD-L1 blockade, CTLA-4 inhibition, radiation therapy and the chemotherapeutic drug temozolomide.^130^ Its penetration of the blood–brain barrier paved the way for the first study of EOS-200271 in patients with WHO Grade IV glioblastoma or WHO Grade III anaplastic gliomas (NCT02764151), and interim data demonstrated that EOS-200271 crossed the blood–brain barrier and was well tolerated at doses up to 500 mg twice daily. However, it lacked efficacy, resulting in Pfizer returning its EOS-200271 rights to iTeos Therapeutics.

#### KHK2455

KHK2455 is an IDO1 inhibitor developed by Kyowa Kirin Pharmaceutical Development, which acts by binding to the haem-free apo-IDO1 protein. KHK2455 has recently entered Phase 1 clinical trials for a host of cancer entities in combination therapy with the anti-PD-L1 monoclonal antibody (mAb) Avelumab in advanced bladder cancer (NCT03915405) and in combination with the anti-CCR4 mAb Mogamulizumab for locally advanced or metastatic solid tumours (NCT02867007).^150^ The clinical trials for KHK2455 have either not started or just started recruiting patients, therefore results are still awaited.

#### LY3381916

This Eli Lily-developed IDO1 inhibitor has entered Phase 1 clinical trials in late stage and metastatic solid tumours either alone or in combination with anti-PD-L1 therapies (NCT03343613). Recently pharmacodynamics data from a preclinical study in solid tumours were released, which revealed that LY3381916 does not bind the mature IDO1 enzyme but rather binds to the haem-binding pocket of the apo-IDO1 protein, thus preventing protein maturation.^151^

#### MK-7162

This IDO1 inhibitor with undefined mechanism of action has been developed by Merck. MK-7162 has entered into Phase 1b clinical trials in combination therapy with anti-PD-L1 treatments in adult participants with advanced metastatic solid tumours (NCT03364049). No results from this trial are in public domain at the moment.

#### NLG802

NLG802, another molecule from NewLink Genetics, is a prodrug of indoximod.^152^ Recently released Phase 1 clinical trial results suggest that NLG802 produced significantly higher pharmacokinetic exposure in advanced solid tumour patients as compared with the molar equivalent of indoximod, while maintaining promising safety parameters (NCT03164603).

### Analysing IDO1 inhibitor clinical trial results: aspects of clinical trials

Given the mostly disappointing results from clinical trials, the question arises as to whether these failures can be attributed to aspects of the particular clinical trials. Crucial aspects of clinical trials include the choice of compound for IDO1 inhibition, dosing, the selection of treatment that IDO1 inhibition is combined with, the selection of cancer type and patient stratification.

#### IDO1 inhibitor choice

**Fig. 2:**
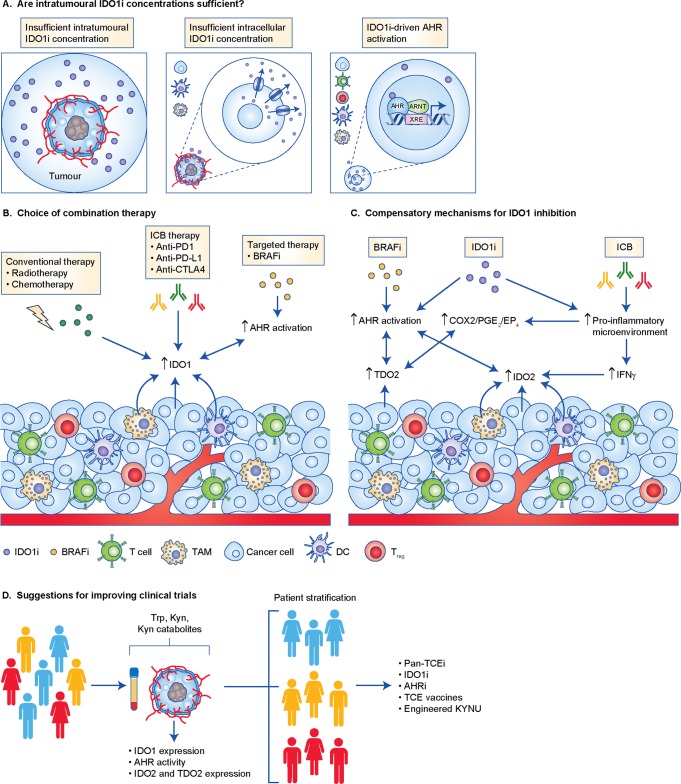
Analysing IDO1 inhibitors clinical trial results. **a** Intra-tumoural penetrance of IDO1 inhibitors (IDO1i) could be hindered due to diminished blood supply to tumours or elevated oncotic pressure in solid tumours (left). Even if IDO1i are able to access tumours, ABC transporters can interfere with their intracellular penetrance (middle). Cellular uptake of IDO1i can lead to AHR activation in various cell types and therefore contribute to cancer progression (right). **b** Combination of IDOi with other types of therapies can lead to the upregulation of IDO1 expression through various mechanisms (see text). In addition, clinical trials have included patients pre-treated with BRAF inhibitors (BRAFi), which were shown to promote an enrichment of a population of cells with constitutive AHR activity. This might further lead to the induction of IDO1 expression and contribute  to  cancer progression. **c** Even if IDO1 inhibition is effectively achieved, IDO2 and TDO2 might compensate for IDO1 blockade. BRAFi and IDO1i can promote AHR activation in various cells present in the tumour microenvironment, which unleashes the intrinsic cancer promoting effects driven by the AHR. Moreover, AHR activation leads to the upregulation of IDO2 and TDO2, which can further activate the AHR and deplete Trp. IDO1i and immune check point blockade (ICB) lead to an increase in cytotoxic TILs, which in turn secrete pro-inflammatory molecules, such as IFN-γ, which also induces IDO2. In addition, the pro-inflammatory microenvironment driven by IDO1i and ICB therapy can also lead to the upregulation of TDO2, via the COX2–PGE_2_–EP4 pathway. **d** In order to obtain a more robust view of the effects of IDO1 inhibition and improve the outcome of its use in clinical trials, we suggest stratifying patients based on the concentration of Trp, Kyn and Kyn-derived metabolites in plasma and tumours, as well as on the expression of the TCE and AHR activity present in tumours. Once stratified, patients can be treated more accurately with one or more therapies targeting Trp catabolism.

Intrinsic properties of the different IDO1 inhibitors, such as activity, intra-tumoural penetrance and off-target effects, can influence their efficacy. A reduction in systemic Kyn levels is only an indirect readout, given that the IDO1 inhibitors are targeting the tumour microenvironment. Tumoural Kyn levels in response to IDO1 inhibition were not routinely monitored in most of the clinical trials. For BMS-986025, the available data show a reduction of up to 90% in tumoural Kyn levels, which appears sufficient to exert biological effects. Such data are not available for epacadostat. Evaluation of the intra-tumoural concentrations of epacadostat would be important, as intra-tumoural penetrance of IDO1 inhibitors could be hindered due to diminished blood supply^143,151^ or elevated oncotic pressure in solid tumours^8,143^ (Fig. 2a, left). Moreover, epacadostat is a substrate of the ABC transporters P-glycoprotein (ABCB1) and the breast-cancer resistance protein (ABCG2),^153^ which are associated with drug resistance and expressed in malignant melanoma^154^ (Fig. 2a, middle). Consequently, intracellular epacadostat concentrations in melanoma could have been low, thus possibly not sufficiently inhibiting IDO1-mediated Trp degradation. Furthermore, IDO1 inhibitors can elicit unfavourable off-target effects, as some clinically tested IDO1 inhibitors have been implicated in activating the AHR,^155,156^ due to their structural resemblance to AHR agonists (Fig. 2a, right). As activation of AHR is one of the main mechanisms mediating immune regulation downstream of IDO1, IDO1-inhibitor-mediated activation of AHR could mitigate the effects of IDO1 inhibition, even if Kyn formation is sufficiently blocked. Furthermore, AHR activation could lead to induction of IDO1/2 and TDO2, as AHR has been demonstrated to be necessary for their upregulation.^75,101,157–159^

#### Dosing, possible effects of pre-treatments and combination therapy

Other reasons for the failure of IDO1 inhibitors could be inappropriate dosing and the selection of the therapy the IDO1 inhibitors were combined with. Diverse treatments such as radiotherapy,^160^ chemotherapy,^161^ demethylating agents^162,163^ and immune checkpoint inhibitors targeting PD-L1 and CTLA-4^19^ have been reported to induce IDO1 expression and/or activity, so doses determined to be sufficient to inhibit IDO1 as a monotherapy might not be sufficient to inhibit the levels of IDO1 in combination therapies (Fig. 2b).

Moreover, IFN-γ, a known inducer of IDO1, can also induce IDO2 expression in cancer cell lines.^65,68^ Because PD-1 and PD-L1 blockade leads to an increase in cytotoxic TILs, which in turn secrete pro-inflammatory molecules, such as IFN-γ,^164,165^ IDO2 expression could be induced in the tumour and its activity might compensate for IDO1 inhibition (Fig. 2c). In a murine model of colon adenocarcinoma, dual inhibition of IDO1 and PD-L1 increased the percentage of intra-tumoural CD4^+^ IFN-γ^+^ and CD8^+^ IFN-γ^+^ T cells, compared with either therapy alone,^19^ suggesting that the increased levels of IFN-γ might mediate induction of IDO2. In agreement, PD-L1 blockade was recently shown to induce IDO1 and IDO2 in a preclinical syngeneic mouse model of sarcoma.^165^ Inhibition of IDO1 might channel Trp towards IDO2. Therefore, when IDO1 is pharmacologically inhibited, IDO2 might become active. Developing inhibitors against IDO2 is challenging as it is hard to express and purify.^166^ Therefore, currently, only few specific IDO2 inhibitors are known, none of which has entered the clinical phase.

Specific to the ECHO-301 trial,^24^ as patients previously having received adjuvant anti-CTLA-4 therapy were included in the study,^24^ it is possible that IDO1 induction by CTLA-4 inhibitors^19^ could have rendered the IDO1 inhibitor concentration insufficient (Fig. 2c). Moreover, this trial included patients pre-treated with BRAFi.^24^ The BRAFi vemurafenib was recently shown to promote an enrichment of dedifferentiated BRAFi persister cells with constitutively active AHR.^66^ As AHR activation is a key effector mechanism of IDO1, the constitutive AHR activity following vemurafenib treatment may counteract the effects of IDO1 inhibition. Moreover, as AHR activation promotes the expression of IDO1/2 and TDO2,^101,157–159^ the IDO1 inhibitor may not have been able to inhibit Trp catabolism due to induction of IDO2 and/or TDO2 (Fig. 2c).

In addition, PGE_2_, a molecule derived from cyclo-oxygenase-1/2 (COX1/2) activity, drives IDO1 and TDO2 expression and activity in human cancer cells through activation of EP4.^35,167^ COX2 is induced in response to inflammatory stimuli, which are present in the tumour microenvironment^168,169^ and enriched upon treatment with immune checkpoint blockade alone^164,165^ and also in combination with IDO1 inhibition.^21^ Therefore, activation of the COX2–PGE_2_–EP4 pathway may have increased IDO1 and TDO2 levels possibly rendering IDO1 inhibitors unable to inhibit Trp degradation (Fig. 2c).

Liu et al.^55^ demonstrated that IDO1 drives PD-1 expression via Kyn-mediated AHR activation. If IDO1 is indeed upstream of PD-1, then IDO1 inhibition might already inhibit PD-1 activity by downregulating its expression thus rendering a synergistic effect of PD-1 and IDO1 inhibition unlikely. Hence, the choice of combination partner is critical for the efficacy of IDO1 inhibition. Furthermore, inhibition of the immune checkpoint components PD-L1 and CTLA-4 when combined with simultaneous IDO1 blockade has recently been reported to result in hepatic injury mediated via immune infiltration in preclinical mouse models.^4^ It remains to be investigated if similar adverse effects also occur in humans.

#### Cancer type and patient stratification

**Fig. 3:**
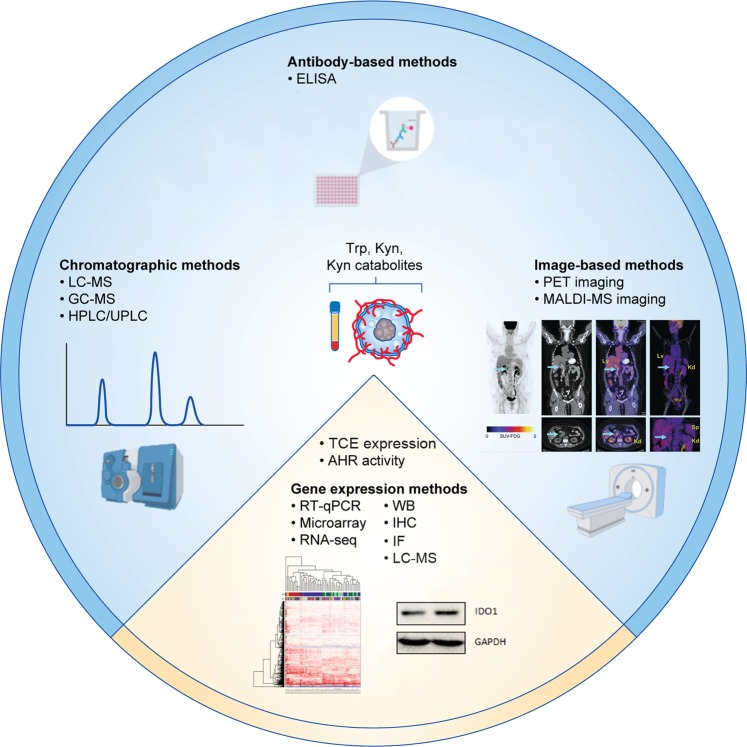
Analytical approaches for quantification expression and activity of tryptophan catabolising enzymes (TCEs) in cancer therapies. The levels of Trp and its metabolites can be measured by chromatographic methods, including high-performance liquid chromatography (HPLC), gas chromatography–mass spectrometry (GC-MS) or liquid chromatography–mass spectrometry (LC-MS).^208^ Antibodies detecting Trp, as well as specific metabolites of the Trp degradation pathway, enable enzyme-linked immunosorbent assay (ELISA) measurements.^163,209–211^ Furthermore, matrix-assisted laser desorption/ionisation (MALDI) mass spectrometry (MS) imaging allows visualisation of Trp and Kyn in tissue,^90,212^ while position-emission tomography (PET) imaging with Trp-derived tracers enables visualisation of Trp uptake in vivo.^213–218^ In addition, TCE expression can be detected on mRNA and protein levels, however care must be taken when selecting anti-IDO1 antibodies, as many lack selectivity, particularly for immunohistochemistry.^27^ The blue area of the circle illustrates methods used for measuring Trp and its metabolites; the yellow area illustrates methods used for detcting the expression of TCE and AHR activity. IF: immunofluorescence; WB: Western blot. PET-imaging example was reproduced under a Creative Commons CC-BY license from.^219^ RNA-seq example was reproduced under a Creative Commons CC-BY license from.^220^ Western Blot example was provided by the authors.

Aside from the IDO1 inhibitors themselves, the type of cancer in which IDO1 inhibition is studied is critical, as IDO1 inhibitors can only be effective if IDO1 is expressed and active. Gliomas, for instance, scarcely express IDO1^7,28^ and IDO1 does not account for the constitutive Trp degradation observed in these tumours.^7^ Therefore, efficacy of IDO1 inhibition by EOS-200271, for example, might not be expected in malignant gliomas. However, radiotherapy and/or PD-1 blockade may induce IDO1, thus resulting in IDO1 inhibition increasing overall survival as shown in a preclinical mouse model. Furthermore, IDO1 expression is very heterogeneous, even within a single cancer entity. Stratification of patients for IDO1 inhibitor treatment based on IDO1 expression and activity (based on Trp and Trp-derived metabolites, such as Kyn and Kyn catabolite levels) in the tumour tissues and body fluids therefore is critical (Fig. 2d). As Trp catabolism in the tumour does not necessarily lead to low Trp levels and Kyn can be further converted into downstream catabolites, measuring additional catabolites downstream of Kyn can provide important information about the efficacy of inhibition of Trp catabolism.^7^ Moreover, as several metabolites downstream of Kyn, such as KynA, XA and CA, are known AHR agonists,^9,75,95,98^ monitoring the concentration of these metabolites is relevant. In support of this approach, recent data showed that PD-L1 blockade in a syngeneic mouse model of sarcoma, induced the expression of several enzymes of the Kyn pathway, including IDO1, IDO2, kynurenine 3-mono-oxygenase (KMO) and kynureninase (KYNU).^165^ This leads to an increased intra-tumoural concentration of Kyn-derived catabolites and a decreased intra-tumoural Kyn/Trp ratio, suggesting that anti-PD-L1 therapy promotes Kyn catabolism.^165^ Unfortunately, stratification of patients to IDO1 inhibitors based on readouts of IDO1 expression and activity have been hardly performed in the clinical trials so far, despite a number of available approaches to assess these parameters (Fig. 3). Furthermore, assessing the activity of GCN2 and AHR pathways in tumour samples could provide additional information for stratifying patients. Most likely, the combined analysis of both enzyme expression and Trp-derived catabolite levels/Trp uptake will be most effective for stratification of patients to inhibitors of Trp catabolism.

### Alternative approaches to target Trp catabolism

As TDO2 catalyses the same reaction as IDO1, its activity might be able to substitute for IDO1 when it is inhibited. Therefore, TDO2 inhibitors and dual IDO1/TDO2 inhibitors have been developed.

### TDO2 inhibitors

TDO2 inhibitors were initially developed as antidepressants to increase systemic Trp levels and thus enhance serotonin concentrations in the brain.^170,171^ Small-molecule inhibitors of TDO2 are being developed for cancer therapy but have not yet reached clinical trials. Patents on TDO2 inhibitors have recently been comprehensively reviewed.^172^

#### Single TDO2 inhibitors

The first TDO2 inhibitor developed, 680C91, also efficiently inhibited TDO2 in glioblastoma cells.^7^ Hsu et al. observed enhanced TDO2 expression in fibroblasts surrounding the implantation of murine lung cancer lines^173^ in a lung cancer model, and treatment with 680C91 resulted in an improved T-cell response, DC function and decreased tumour metastasis.^173^ However, 680C91 is poorly soluble in aqueous solutions and shows low oral bioavailability, limiting its in vivo use.^170^ In addition, 680C91 activates AHR target gene expression (Opitz, unpublished observation), suggesting that it might act as an AHR agonist, thus further limiting its usefulness in cancer therapy, as will be described below. LM10, a TDO2 inhibitor with improved solubility and bioavailability,^174^ was used to show that TDO2 inhibits anti-tumour immune responses and promotes tumour growth in a murine cancer model.^15^ Other single TDO2 inhibitors are also under development, several of these such as fused imidazo-indoles-based inhibitors developed by RedX Pharma (Patent WO 2016/051181, Patent WO 2016/059412) and indazole-based TDO2 inhibitors developed by IOmet Pharma (Patent WO 2016/071283, Patent WO 2015/150097) are currently in various stages of experimental or preclinical studies.

#### Dual IDO1–TDO2 inhibition

On the basis of the co-expression of IDO1 and TDO2 in several cancer types and the potential for TDO2 to undergo compensatory upregulation in response to IDO1 inhibition, combining IDO1 inhibitors with TDO2 inhibitors or using dual IDO1–TDO2 inhibitors might provide an effective means of targeting Trp catabolism. Accordingly, several dual inhibitors, such as HTI-1090 and DN1406131, are currently in various stages of development. Significantly reduced Kyn levels in response to the IDO1–TDO2 inhibitor RG70099 have been documented in preclinical cancer models,^175^ while reduced tumour volumes in response to the IDO1/TDO2 inhibitor EPL-1410 have been reported in preclinical trials.^176^ The IDO1/TDO2 dual inhibitors CB548 and CMG017 were recently described to elicit a robust anti-cancer immune effect and to synergistically inhibit cancer progression in combination with an immune checkpoint inhibitor.^177^ A further effort towards creating dual IDO1/TDO2 inhibitors as well as pan dioxygenase inhibitors that also target IDO2 has been reported.^154,178^ However, it is currently unknown how well such inhibitors would be tolerated, as even though mice seem to have more TDO2 expression in liver than humans,^179^ *Tdo2*^*–/–*^ mice have extremely high systemic levels of Trp,^70,71,180^ which affect Trp metabolism along the Kyn pathway^180^ and might cause shunting of Trp into other pathways such as serotonin, or tryptamine formation. Moreover, Trp degradation down the Kyn pathway leads to the de novo synthesis of NAD^+^, particularly in the liver.^181^ Inhibition of TDO2 or dual inhibition of IDO1 and TDO2 might therefore reduce NAD^+^ levels. NAD^+^ plays a central role as a coenzyme in metabolism and redox reactions, in the removal of oxidative DNA damage by NAD^+^-dependent poly(ADP ribose) polymerases (PARPs) and in transcriptional regulation mediated through the action of NAD^+^-dependent sirtuins.^182^ Although no hepatic tumorigenicity has been described in *Tdo2*^–/–^ mice so far, reduced expression of *Tdo2* and consequent downstream Kyn-pathway enzymes has been reported to promote hepatic tumorigenesis through DNA damage by decreasing liver NAD^+^ levels.^183^ It therefore remains to be determined how well dual IDO1–TDO2 inhibition is tolerated.

### Alternative approaches beyond small-molecule inhibition of dioxygenases

#### Vaccines against Trp catabolic enzymes

Although inhibition of IDO1/2 and TDO2 enzymatic activity has been the mainstay of efforts targeting these enzymes, alternative strategies have also been explored. One approach is based on T-cell reactivity against IDO1, IDO2 and TDO identified in cancer patients^184–187^ The detection of T-cell responses against IDO1 has sparked small Phase 1 and Phase 2 clinical trials testing vaccination with a synthetic IDO1 peptide either alone,^188^ in combination with the anti-CTLA-4 mAb ipilimumab^189^ or in combination with a vaccine against a survivin peptide and the chemotherapeutic agent temozolomide.^190^ A Phase 1 trial in stage III–IV NSCLC patients treated monthly with an IDO1 peptide vaccine alone, reported a 6-year overall survival of 20%.^191^ A recently developed technology termed T-win® aims to activate immune cells against both Tregs as well as malignant cells by vaccination against long peptide epitopes of IDO1 and/or TDO2.^14^ Furthermore, T-win® has also been proposed to target TAMs and revert them back to an M1 phenotype by converting the immunosuppressive tumour microenvironment into a pro-inflammatory one.^17^

#### Engineered KYNU

Another approach to limit the immunosuppressive and cancer-promoting effects of IDO1-mediated and/or TDO2-mediated Kyn formation is depletion of extracellular Kyn by engineered KYNU. Single doses of engineered KYNU potently reduced Kyn concentrations and increased the levels of CD8^+^ T cells in murine tumour models.^192,193^ KYNU converts Kyn into anthranilic acid (AA). Little information is available about the uptake of AA, although it is known to cross the blood–brain barrier and might contribute significantly to the cerebral pools of this metabolite.^194^ Although the effects of AA accumulation are unknown, it is taken up by cells and might be converted further into hydroxyanthranilic acid and the AHR agonist, CA.^95^ Given that many of the metabolites downstream of KYNU are also potent AHR ligands, depletion of the KYNU substrate, Kyn, might not be sufficient to exert the desired effects. Therefore, further studies are required to determine the tolerability and efficacy of engineered KYNU.

#### AHR inhibition to block the effects of enhanced Trp degradation in cancer

AHR expression is increased in aggressive malignancies and constitutively localises to the nucleus, suggesting that it is chronically activated to facilitate tumour progression.^195,196^ Moreover, AHR antagonism diminished cell viability in patient-derived glioma and meningioma cells following in vitro drug treatments,^195^ unlike inhibition of Trp-degrading enzymes. Therapeutic modulation of AHR is therefore currently being explored for cancer therapy.^196^ AHR inhibition represents a strategy to tackle immunosuppression and cancer cell intrinsic malignant properties mediated by Trp catabolites regardless of the enzymes involved in their formation. In support of this approach, a recent study showed that AHR inhibition elicited anti-tumour activity alone and further increased efficacy when combined with gemcitabine or PD-L1 blockade in diverse syngeneic mouse tumour models.^197^ Furthermore, by inhibiting the expression of IDO1, IDO2 and TDO2,^33,34,75,157^ AHR antagonism might even prevent effects of these enzymes that are independent of downstream AHR activation. Currently, the AHR inhibitors CH223191 and StemRegenin 1 (SR1) are commonly used to study AHR-activation mediated effects.^1,55,66,97,198–200^ CH223191 and SR1 exert their inhibitory effect through direct binding to AHR.^201,202^ AHR inhibitors are currently being developed by multiple pharmaceutical companies including Kyn Therapeutics,^203^ Hercules Pharmaceuticals,^103^ Phenex Pharmaceuticals,^197^ Ideaya Biosciences^204^ and Bayer AG.^205–207^ The AHR inhibitor HP163 (Hercules Pharmaceuticals), reduced the number of immunosuppressive CD11b^+^PD-L1^+^ or CD11b^+^CCR2^+^ cells in tumour-draining lymph nodes in a mouse model of oral cancer and reduced the tumour growth in immunocompetent orthotopic models of different types of cancer.^103^ The inhibitor from Ideaya Biosciences, IDE-AhRi, was recently shown to suppress the polarisation and immunosuppressive activity of M2 macrophages.^204^ Furthermore, the AHR inhibitor BAY-218 (Bayer AG) stimulated pro-inflammatory monocyte and T-cell responses in vitro and promoted anti-tumour immune responses and reduced tumour growth in the syngeneic mouse tumour models CT26 and B16-OVA. Notably, BAY-218 enhanced therapeutic efficacy of PD-L1 blockade in the CT26 model.^205^ This compound also recently entered a Phase 1 clinical trial in patients with advanced cancer.^207^ Although initial preclinical results look promising, only clinical trials will show how well AHR inhibitors are tolerated, whether they effectively inhibit tumour progression, and in which cancer entities they are effective.

## Concluding remarks

Based on its immunosuppressive and cancer-promoting effects, Trp degradation remains an important target in immuno-oncology. In future clinical trials, it will be critical to stratify patients to inhibitors of Trp catabolism based on enzyme expression, Trp catabolite levels and the activation of downstream signalling pathways, as only those patients can benefit whose tumours indeed show expression and activity of Trp-catabolising enzymes. Collection of tumour tissue and biological fluids throughout treatment will be essential to monitor the efficacy of enzyme inhibition. Possible effects of pre-treatments and combination therapies on the inhibition of Trp degradation should be taken into account and closely monitored. IDO2 and TDO2 may compensate for IDO1 inhibition. Pan-Trp degradation inhibitors may circumvent this problem, but their safety and tolerability remain to be investigated. Vaccination against Trp-degrading enzymes and removal of Trp catabolites, e.g. by engineered KYNU, represent additional interesting approaches for cancer therapy. Furthermore, strategies targeting AHR activity might be beneficial in tackling immunosuppression and malignant properties of cancer cells mediated by Trp catabolites. By inhibiting the expression of IDO1/2 and TDO2, AHR inhibition might even prevent effects of these enzymes that are independent of downstream AHR activation. Subsequent to this audit of potential causes for the failure of IDO1 inhibition in recent clinical trials, we are hopeful that pharmaceutical companies will not abandon the entire concept of inhibition of Trp catabolism, but rather closely analyse the data that have been generated such that future clinical trials can overcome these obstacles.

## Data Availability

The data from The Cancer Genome Atlas (TCGA) and Genotype-Tissue Expression (GTEx) Project is publicly available.
